# Evaluation of 3D Markerless Motion Capture System Accuracy during Skate Skiing on a Treadmill

**DOI:** 10.3390/bioengineering11020136

**Published:** 2024-01-29

**Authors:** Petra Torvinen, Keijo S. Ruotsalainen, Shuang Zhao, Neil Cronin, Olli Ohtonen, Vesa Linnamo

**Affiliations:** Faculty of Sport and Health Sciences, University of Jyväskylä, 88610 Jyväskylä, Finland; keijo.s.ruotsalainen@jyu.fi (K.S.R.); zhaoshuangzs@hotmail.com (S.Z.); neil.j.cronin@jyu.fi (N.C.); olli.ohtonen@jyu.fi (O.O.); vesa.linnamo@jyu.fi (V.L.)

**Keywords:** kinematics, motion analysis, artificial intelligence, treadmill skiing, markerless motion capture

## Abstract

In this study, we developed a deep learning-based 3D markerless motion capture system for skate skiing on a treadmill and evaluated its accuracy against marker-based motion capture during G1 and G3 skating techniques. Participants performed roller skiing trials on a skiing treadmill. Trials were recorded with two synchronized video cameras (100 Hz). We then trained a custom model using DeepLabCut, and the skiing movements were analyzed using both DeepLabCut-based markerless motion capture and marker-based motion capture systems. We statistically compared joint centers and joint vector angles between the methods. The results demonstrated a high level of agreement for joint vector angles, with mean differences ranging from −2.47° to 3.69°. For joint center positions and toe placements, mean differences ranged from 24.0 to 40.8 mm. This level of accuracy suggests that our markerless approach could be useful as a skiing coaching tool. The method presents interesting opportunities for capturing and extracting value from large amounts of data without the need for markers attached to the skier and expensive cameras.

## 1. Introduction

Marker-based motion capture systems have been used extensively in biomechanics research. Infrared marker-based systems are considered an accurate method, but with some challenges and disadvantages. Traditionally, marker-based systems are time-consuming, often limited to laboratory environments, and require expensive equipment and high-level expertise. It is challenging or impossible to use marker-based motion capture systems in several cases, for example, in sports competitions or games. There are also challenges related to the use of markers, such as soft tissue artifact and marker placement errors [1,2,3]. Moreover, marker-based systems show errors when compared with biplanar videoradiography [1,2]. For example, root mean squared error (RMSE) angular differences ranging between 1.06° and 8.31° have been observed during walking and running [2].

Deep learning-based human pose estimation methods have been extensively studied in the computer vision literature, especially during the past 10 years [4], and this provides opportunities for applying these methods to multiple use cases like markerless motion capture. Nowadays, several deep learning-based markerless methods provide opportunities for detecting human movements with moderate accuracy [5,6]. Markerless methods can facilitate the understanding of human movement in different contexts, for example, in sports biomechanics, sports medicine, injury risk assessment, and rehabilitation [7,8]. There are several open-source pose estimation algorithms available such as OpenPose [9] and DeepLabCut [10], and also some commercial products such as Theia3D (Theia Markerless Inc., Kingston, ON, Canada) and Microsoft Kinect V2. Naturally, there are some challenges with markerless methods, such as mistakes in manual labelling during model training and issues related to the training data quantity and quality [11].

Several studies have used markerless open-source deep learning-based methods to perform 2D kinematic analyses using a single-camera view [12,13,14,15]. For example, high Pearson correlations (>0.97) were found for shoulder and knee angles during static hold arm-raises and squats when evaluated against marker-based motion capture [14]. With multiple calibrated cameras, 2D open-source pose estimation methods can be used to successfully detect the 3D position of human joint centers, joint angles, and segment angles [16,17,18,19,20]. When compared with marker-based motion capture data, differences in joint center locations were approximately 15–50 mm for activities such as throwing, walking, running, and jumping [16,17,18]. Some differences were also found between different pose estimation algorithms such as OpenPose, AlphaPose, and DeepLabCut during walking, running, and jumping [17]. The characteristics of markerless video cameras were also found to have small effects on the results [16]. In some studies, lower limb joint angles and segment angles were primarily calculated when evaluating markerless motion analyses against marker-based systems [18,19]. In a fully automated 3D markerless motion capture workflow with OpenPose, lower limb joint angles were detected with mean differences ranging between −3.9° and 10.5°, and RMSEs ranging between 1.2° and 7.4° for hip, knee, and ankle joint rotations during jumping, walking, and running [19]. In a clinical approach, lower limb joint angles were detected with an RMSE ranging between 3.16° and 11.90° [18]. In addition, studies using the commercial markerless product Theia3D obtained quite similar results than with open-source methods [21,22,23]. For example, RMSDs were less than 2.5 cm for joint centers except the hip, and less than 5.5° for segment angles when comparing Theia3D markerless to marker-based motion capture during treadmill walking [22].

Cross-country skiing and especially skate skiing is a complex movement involving both lower and upper body muscles and skiing equipment. To date, studies have focused on the biomechanics of skiing when skiers need to quickly modify skiing techniques at different speeds and on different terrains [24]. It would be of interest to study whether a markerless system would be accurate enough to analyze and provide feedback on a skier’s skiing technique.

This study presents a DeepLabCut-based method that can be used for the 3D kinematic analysis of skate skiing on a treadmill. The method was used to determine joint centers and joint angles. The aim of this study was to compare our markerless motion capture system with a marker-based system in terms of skate skiing on a treadmill. It was hypothesized that our markerless motion capture system would be able to detect the joint centers and joint angles with about the same level of accuracy as previous similar studies, i.e., mean differences less than 50 mm for joint centers and mean differences between −5° and 5° for joint angles.

## 2. Materials and Methods

### 2.1. Participants and Experimental Protocol

Data from 13 female [Age: 21.7 ± 4.6 years] and 19 male [Age: 20.6 ± 7.5 years] athletes roller skiing with the G3 skating technique on a skiing treadmill were used to train the markerless model. The athletes were part of the Finnish junior national teams in cross-country skiing, biathlon and Nordic combined, and some were part of Vuokatti-Ruka sport academy. The athletes provided their consent to use their skiing videos in the development of the markerless models. Training data were collected during the athletes’ normal training sessions. The angle of the skiing treadmill ranged between two and three degrees, and the speed ranged between 12 and 30 km per hour in the training sessions. Two 20 s videos were recorded with two cameras from each athlete.

A total of 10 experienced skiers who were familiar with roller skiing on a skiing treadmill (5 females [Age: 21.0 ± 3.1 years; height: 1.73 ± 0.49 m; weight: 63.9 ± 7.3 kg] and 5 males [Age: 24.4 ± 10.7 years; height: 1.85 ± 0.69 m; weight: 81.0 ± 4.1 kg]) volunteered in the evaluation study and provided written informed consent. The participants used the same pair of roller skis (Marwe, Skating 620 XC, wheel No. 0) and poles of suitable length (Swix, Triac 3.0). Each participant had a single testing session, which included a static calibration where the participant stood still on the skiing treadmill to make sure that all markers were visible, and that the systems were working correctly. Then, the participant performed four roller skiing trials with the G1 skating technique (two for both sides), and three roller skiing trials with the G3 skating technique. The G1 skating technique is “a skating form of the diagonal stride technique of classic skiing, in which the leg and arm are employed diagonally, with a single pole-push with each contra-lateral leg-push in a skating-like fashion” [25]. With the G3 skating technique, the skier performs “one double-pole together with every leg push” [25]. Each roller skiing trial started when the skier struck the treadmill with the ski pole to enable the systems to be synchronized, and then included at least thirty seconds of skiing. G1 trials included about 20 skiing cycles, and G3 trials included at least 15 skiing cycles. A skate skiing cycle includes each leg pushing once. A G1 cycle includes one impact of the poles, and G3 cycle includes two impacts of the poles. The roller skiing trial protocol is shown in Table 1.

### 2.2. Motion Capture Systems and Laboratory Configuration

**Figure 1:**
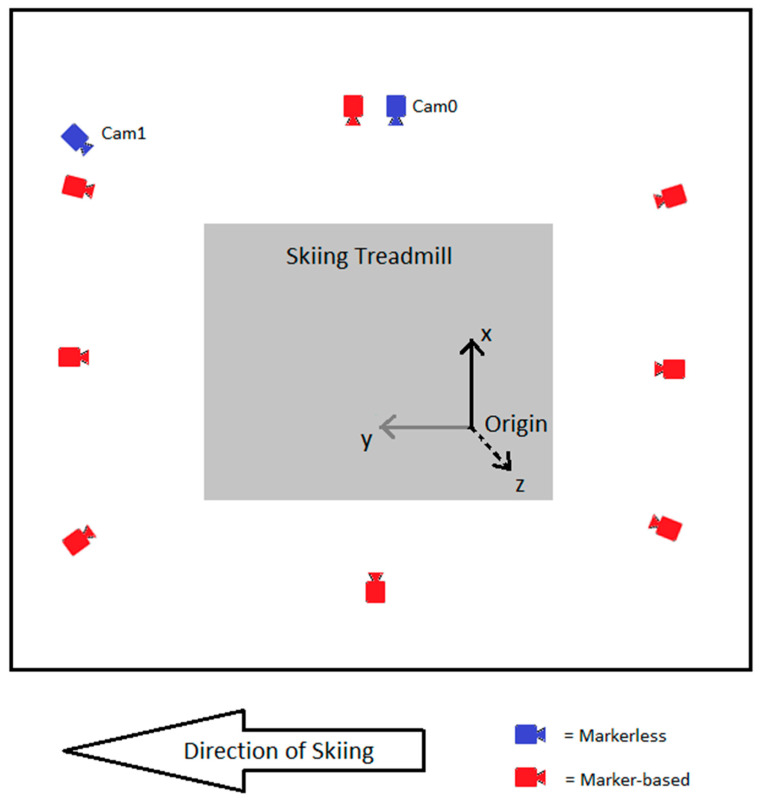
Skiing Laboratory configuration, camera placements, and coordinate system configuration.

This study was conducted at the Skiing Laboratory of the University of Jyväskylä in Vuokatti, which is equipped with a 2.7 m × 3.5 m skiing treadmill (RL3500E, Rodby Innovation AB, Vänge, Sweden). In this study, we used two motion capture systems concurrently. Marker-based data were captured using an infrared 8-camera marker-based motion capture system (100 Hz, Vicon, Oxford, UK) and NEXUS 2.8.1 software (Vicon, Oxford, UK). The cameras were positioned around the skiing treadmill on the ceiling at a height of about 2.5 m. In addition, the Skiing Laboratory was equipped with video cameras (LILIN, Taiwei, New Taipei City, Taiwan) and a Coachtech online measurement and feedback system [26]. In this study, two video cameras capable of capturing 1280 × 720 resolution videos at 100 Hz were used for markerless motion capture. Videos were recorded using the Coachtech system. A sampling frequency of 100 Hz was used in both motion capture systems, as this was the maximum rate possible for the video cameras. The video cameras were positioned to the right side (Cam0, perpendicular to skiing direction) and right obliquely (Cam1, 45 degrees to skiing direction) from the skiing treadmill. Cam0 was at a height of 1.05 m and a distance of 2.00 m from the skiing treadmill. Cam1 was at a height of 2.5 m and a distance of 1.90 m from the skiing treadmill. The Skiing Laboratory configuration and both system’s camera placements are shown in Figure 1.

### 2.3. Markerless Model Development

**Figure 2:**
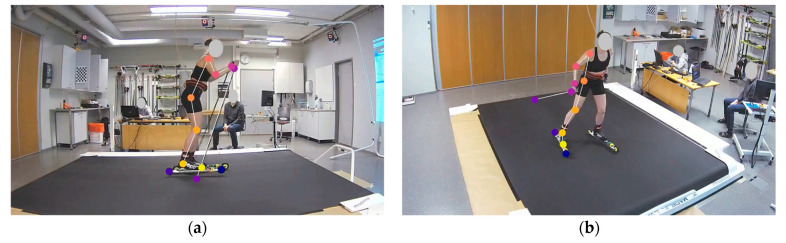
Examples of images labeled by our 2D models: (**a**) from Cam0 and (**b**) from Cam1.

A total of 64 videos from both markerless video cameras were used in the development of the markerless model. Markerless 3D models were developed using DeepLabCut, which is an open-source pose estimation method based on transfer learning with deep neural networks [10]. Two-dimensional models were trained for both video cameras separately using DeepLabCut, and pixel coordinates were later transformed to global 3D coordinates using the Direct Linear Transformation (DLT) algorithm [27]. Training images were selected randomly from the videos using DeepLabCut’s frame extraction method and imgaug algorithm. From each video, 15–20 images were selected, and about 1000 images per camera were used to train each 2D model. The images were manually labeled using DeepLabCut’s graphical user interface. Manually labeled markers were placed on the joint centers of the wrist, elbow, shoulder, hip, knee, and ankle. In addition, the tip of the skiing boot was labeled to reflect toe position. In the 2D models, only the right side of the skier was detected, as well as the right pole and right roller ski. Examples of the output of both 2D models are shown in Figure 2.

The labeled images were then used to train deep neural networks. Labeled images were collected and divided randomly into a 95% training and 5% test split using DeepLabCut’s training set creation method and imgaug algorithm. DeepLabCut’s default network configuration parameters were used. ResNet50 models for both video cameras were initialized with weights trained on ImageNet, and the cross-entropy loss between the predicted score map and the ground truth score map was minimized using stochastic gradient descent. The deep neural networks were trained with 700,000 iterations, and after training, the 2D models were evaluated using the 5% test split. The mean training error was 1.80 pixels (approximately 0.61 cm), and the mean test error was 2.27 pixels (approximately 0.77 cm) for the model of Cam0. The mean training error was 2.10 pixels (approximately 0.72 cm), and the mean test error was 2.63 pixels (approximately 0.89 cm) for the model of Cam1.

### 2.4. System Calibration Procedure

Both motion capture systems were calibrated before the measurements. The same origin and axis directions were used for both systems. The configuration of the coordinate system is shown in Figure 1. The origin was defined at the left side of the skiing treadmill. The x-axis (black in Figure 1) was defined as the latitudinal axis of the treadmill, the y-axis (gray in Figure 1) as the longitudinal axis of the treadmill, and the z-axis (black dashed line in Figure 1) as perpendicular to the treadmill, pointing upward.

**Figure 3:**
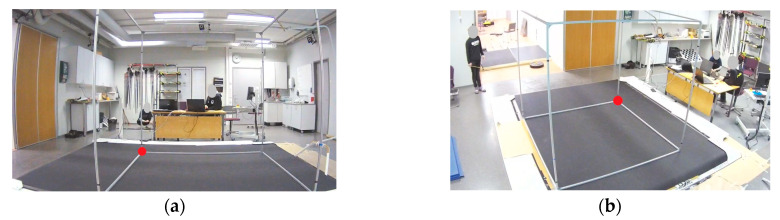
Placements of the calibration cube and origin (red circle) in the Skiing Laboratory: (**a**) from Cam0 and (**b**) from Cam1.

With the markerless system, a calibration procedure was performed using an aluminum calibration cube that was 2 m long in each dimension. The calibration cube was placed on the skiing treadmill with one corner placed at the origin. The placements of the calibration cube and origin are shown in Figure 3. Eight points were selected, marked, and measured from the calibration cube. The selected points were used as calibration points resulting in eight known 3D coordinates. These calibration points were manually digitized from both markerless video cameras. Using these calibration coordinates, both video camera’s x- and y-coordinates were transformed to global 3D coordinates using the DLT algorithm [27]. 

For the marker-based system, the coordinate system was defined by placing a Vicon L-Frame on the treadmill, allowing the coordinate system origin and axis directions to be defined. The marker-based system calibration was performed using Vicon NEXUS 2.8.1 software.

### 2.5. Data Collecting and Processing

In the evaluation study, markerless data were captured using the Coachtech system with two video cameras. Videos were analyzed using the developed 2D models, and markerless data were filtered using a 4th order low-pass Butterworth filter (cut-off frequency, 12 Hz). The synchronization of the videos was ensured using the strokes of the pole on the treadmill from the beginning of each trial. The pixel coordinates were transformed to global 3D coordinates with the DLT algorithm. These 3D coordinates were further filtered with a 4th order low-pass Butterworth filter (cut-off frequency, 6 Hz). The filtering and coordinate transformation were performed using Matlab (v2021b, MathWorks, Inc., Natick, MA, USA).

**Figure 4:**
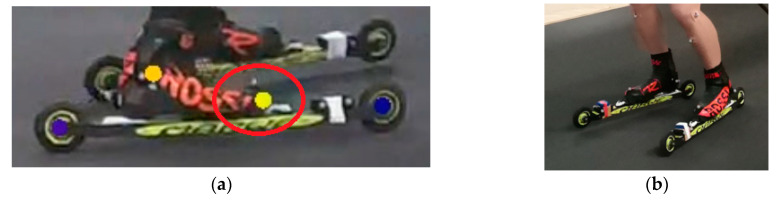
(**a**) Toe marker placements for both the markerless and marker-based systems are shown in the red circle. The markerless toe marker is indicated by the yellow circle, and the marker-based marker appears as a gray circle. (**b**) Marker-based toe marker placement viewed from a different direction.

Marker-based data were captured using a modified marker set comprising 39 individual passive reflective markers on the participant and 12 markers on the skiing equipment: three markers on both roller skis and three markers on both poles. The marker set is shown in Appendix A. The recorded data were labeled and visually verified using Vicon NEXUS 2.8.1 software. Marker trajectory data gap filling and marker-based data filtering with a 4th order low-pass Butterworth filter (cut-off frequency, 6 Hz) were performed using Vicon NEXUS 2.8.1 software. The joint centers of the wrist, elbow, knee, and ankle were computed from the mid-points of the lateral and medial markers. The shoulder joint center was computed from the mid-points of the anterior and posterior shoulder markers. The hip joint center was calculated using the method described by Bell et al. (1989) [28]. Toe markers were placed on top of the skiing boots due to the movement of the skiing boots and roller skis during skiing, so the toe marker positions slightly differed from the points detected by the markerless system. The toe marker placements for both systems are shown Figure 4. Static calibrations were used to determine the difference in each participant’s right toe placement between systems, and this was considered before statistical analysis. The joint center calculation and defining displacements of the right toe placement between systems were performed using Matlab. 

From each skiing trial, 10 skiing cycles were selected and time normalized. The skiing cycles were defined using the right pole markers. The start of each skiing cycle was defined as the point at which the right pole touched the treadmill. To compare the performance of the markerless and marker-based systems, the 3D Euclidean distances of each right-side joint center and toe placement at each time point were calculated. In addition, the vector angles at the elbow, shoulder, hip, knee, and ankle were calculated from both systems to describe the movements of skiing. The elbow angle was defined as the supplement of the angle between the two 3D vectors connecting the elbow position to the wrist and shoulder positions. The hip angle was defined as the supplement of the angle between the two 3D vectors connecting the hip position to the shoulder and knee positions. The knee angle was defined as the supplement of the angle between the two 3D vectors connecting the knee position to the hip and ankle positions. These are thus a simplified proxy for the elbow, hip, and knee flexion angles. The shoulder angle was defined as the angle between the two vectors connecting the shoulder position to the elbow and hip positions. The shoulder angle thus reflects the angle between the upper arm and the upper body. The ankle angle was defined as the complement of the angle between the two vectors connecting the ankle position to the knee and toe positions. The ankle angle is thus a simplified proxy for the ankle plantar/dorsiflexion angle. All angles were calculated using Matlab.

### 2.6. Statistical Analysis

The systems were compared using Bland–Altman analyses [29]. For the joint centers and toe placements, the mean difference (bias), standard deviation (SD) and 95% limits of agreement (LoA) were calculated with a one-sample *t*-test. For the vector angles, the bias, SD, and LoA were calculated with a paired-sample *t*-test. Additionally, for the vector angles, the root mean squared errors (RMSE), Pearson correlations (r), and intra-class correlation coefficient (ICC) were computed to indicate validity between systems. For the Pearson correlations, coefficients were interpreted with values of <0.30, 0.30–0.50, 0.50–0.70, 0.70–0.90, and >0.90 representing negligent, low, moderate, high, and very high correlations, respectively [30]. The ICCs for absolute agreement were estimated based on a single measurement and two-way mixed effects model. The ICCs were used to indicate the agreement, with values of <0.50, 0.50–0.75, 0.75–0.90, and >0.90 representing the quality thresholds for poor, moderate, good, and excellent validity, respectively [31]. The statistical analyses were performed using SPSS 28.0.1.1 (IBM Corporation, Armonk, NY, USA), and the significance level was set at *p* ≤ 0.05.

## 3. Results

A total of 70 trials with 700 skiing cycles were compared between the markerless and marker-based systems: 40 trials with the G1 technique and 30 trials with the G3 technique. The results from the participants’ right sides are reported below.

### 3.1. Joint Centers and Toe Placements

The results of the comparisons between the systems for joint centers and toe placements are shown in Table 2.

For all participants, the mean difference ranged from 24 to 41 mm at the wrist, elbow, shoulder, hip, knee, and ankle joint centers and toe positions. For both techniques, the smallest systematic differences and LoA were observed at the shoulder joint center (G1: 26.6 mm; G3: 24.0 mm) with standard deviations of 19.7 mm (G1) and 14.1 mm (G3). The wrist joint center and toe placement displayed the largest systematic differences with the highest standard deviation and LoA with both techniques. The elbow, hip, knee, and ankle joint center mean differences were typically larger than those at the shoulder but smaller than that at the wrist or toe with their standard deviation and LoA following the same trend.

**Figure 5:**
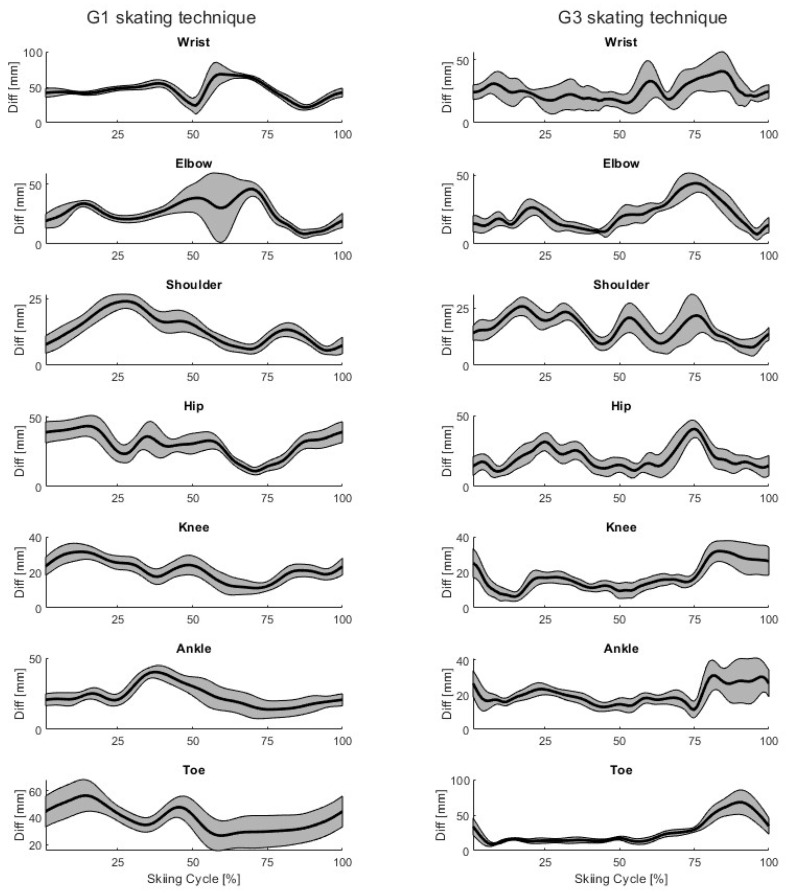
Example mean ± SD differences between markerless and marker-based joint centers and toe placements of a single participant (P03) during G1 and G3 skating technique cycles. The mean difference is shown as a black line, and the standard deviation as the shaded gray area.

For both techniques, the mean (black) ± SD (gray, shaded area) time-series differences in the joint centers and toe placements are shown for a single participant in Figure 5. Two trials were selected from both techniques, so 20 time-normalized skiing cycles are included for each.

### 3.2. Joint Vector Angles

The results of the comparisons between the systems for joint vector angles are shown in Table 3.

Good levels of agreement were observed between the markerless and marker-based results. The Pearson correlations showed high to very high agreement between systems with the highest agreement in the elbow (0.97) and hip (0.97) angles for the G3 technique. The lowest Pearson correlations were observed for ankle angles with r-values of 0.82 and 0.84. The Pearson correlations were statistically significant (*p* < 0.001) for all joint vector angles and for both techniques. The ICC values also showed good to excellent validity between systems with the highest agreements in the elbow (0.97), shoulder (0.97), and hip (0.97) angles for the G3 technique. The lowest ICCs were observed for ankle angles with values of 0.80 and 0.77.

The mean differences in joint vector angles ranged between −2.47 and 3.69 degrees. The lowest systematic differences were observed at the elbow (<1°), but the standard deviation was the largest (>5°). For hip and knee angles, good agreement was observed with low mean differences (<1.1°) and a low standard deviation (<4°). The largest mean differences were observed for shoulder angles, most notably with the G1 technique with a mean difference and standard deviation of 3.69° ± 3.84°.

RMSE values ranged between 3.01 and 7.23 degrees for the joint vector angles. The lowest RMSE values were observed at the knee (<3.5°). For hip and knee angles, quite good agreement was observed with RMSE values < 5°. The largest RMSE values were observed for elbow angles (>5°).

**Figure 6:**
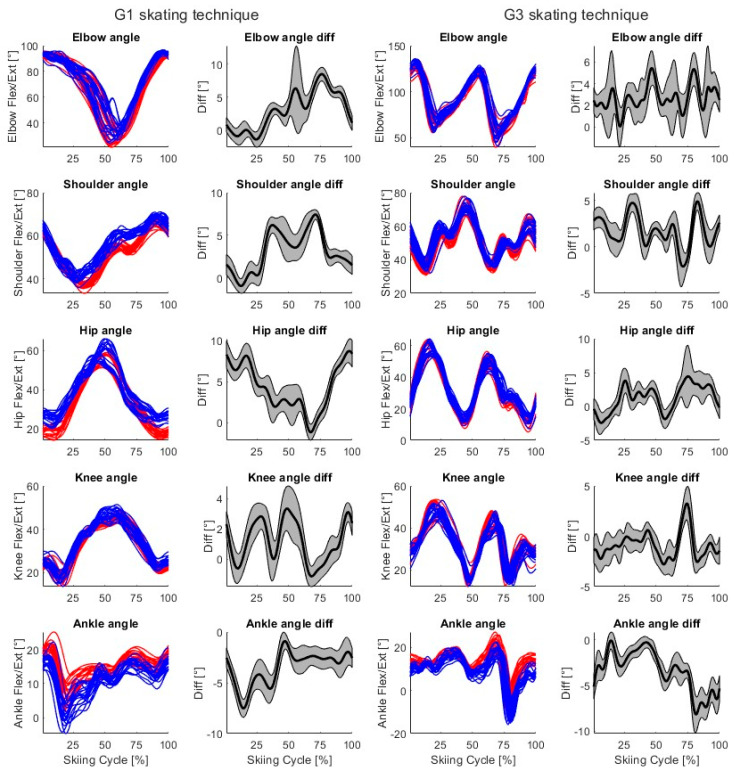
Columns 1 and 3—Example time-normalized markerless (blue) and marker-based (red) joint vector angles for a single participant (P03) during G1 and G3 skating technique cycles. Columns 2 and 4—Mean (black) and ± SD (gray, shaded area) differences for each calculated joint vector angle during G1 and G3 skating technique cycles. For both skating techniques, two skiing trials are included, so 20 skiing cycles are visualized.

For both skating techniques, time-normalized markerless (blue) and marker-based (red) joint vector angles and mean (black) ± SD (gray, shaded area) time-series differences for a single participant (P03) from a single skiing trial are shown in Figure 6. For both techniques, two trials and 20 skiing cycles are included.

## 4. Discussion

This study presents an evaluation of a 3D markerless motion capture system for skate skiing on a treadmill, focusing on the skier’s right-side joint centers and joint vector angles. Our markerless motion capture system was able to detect 3D kinematics with a good level of agreement relative to the marker-based motion analysis, most notably for the joint vector angles (Table 3).

Our markerless motion capture system can correctly detect joint centers during skate skiing (Table 2). The mean differences and standard deviations were generally lower with the G3 skating technique compared to with the G1 technique, which is probably because the markerless model was developed using videos from the G3 skating technique. It is noteworthy that during the skiing cycle, the accuracy of the markerless method varied slightly, even for the same participant (Figure 5). These results are in line with earlier studies, where the joint centers were detected using open-source markerless methods and marker-based systems during throwing, jumping, walking, and running [16,17,18]. Quite similar results were observed in an earlier study in detecting joint centers using OpenPose, AlphaPose, and DeepLabCut during walking, running, and jumping, with mean differences between 14 and 58 mm [17]. Similar results were also obtained in another previous study, where joint centers were detected using OpenPose with multiple synchronized video cameras during walking, countermovement jumping, and ball throwing, with mean absolute errors (MAE) of less than 30 mm in 80% of the trials [16]. Compared to a commercial markerless product, Theia3D, the results of joint center detection are also quite similar to those obtained with open-source markerless methods. For example, Theia3D detected lower limb joint centers with a root mean square of the 3D distances (RMSD) between 20 and 30 mm during countermovement jumping [21]. In another study, Theia3D detected lower and upper limb joint centers with average 3D Euclidean distances between 11 and 36 mm [22].

In the present study, the elbow, shoulder, hip, and knee joint vector angles consistently demonstrated very high levels of agreement between the markerless and marker-based systems, while the ankle joint vector angles demonstrated high levels of agreement (Table 3). These findings demonstrate that our 3D markerless motion capture system can correctly reproduce the movements of skate skiing. Additionally, the calculated joint vector angles differ slightly between systems during the skiing cycle (Figure 6), because joint center detection varies between systems (Figure 5). Our joint angle results are quite similar to those of previous studies using open-source markerless methods and commercial markerless products [19,23,32]. For example, joint angles have been detected using OpenPose during jumping, walking, and running with higher mean differences (between −3.9° and 10.5°) but lower standard deviations (between 1.8° and 4.4°) than in this study [19]. In another study, lower limb joint angles during gait in clinical patients were detected using Theia3D with RMSDs generally less than 6° [23]. Upper limb joint angles have been detected during static postures and basic movements (holding boxes) using Microsoft Kinect V2 with MAEs from 2.5° to 12.9° and using the Captiv system (L7000) with MAEs from 1.9° to 14.9° when compared to a goniometer [32]. The results of our study are therefore in line with those of previous studies related to open-source markerless methods and commercial markerless products.

The results of this evaluation study are highly promising, and the differences between systems typically fell within the known uncertainties of marker-based motion capture. The development process of the markerless model influences the system’s accuracy because the training images were labeled by a human and may thus include errors of judgement. In addition, the number, placements, and characteristics of the video cameras can affect the accuracy of a markerless motion capture system. However, marker-based systems also have inherent errors, which are mainly due to skin artifact. In previous studies, errors of up to ~8° have been reported when comparing marker-based motion capture to biplanar videoradiography for ankle joint rotations during walking and running [2], and knee flexion/extension angles during jumping [1]. Additionally, one previous study reported marker displacements of up to ~20 mm due to the effects of soft tissue artefacts at the knee during stair ascent using 3D fluoroscopy and a marker-based system [3]. It is noteworthy that the accuracies of marker-based systems also vary during the detected cycles (walking, running, jumping, and throwing) [1,2,3].

The differences observed in this study generally fall within the known challenges of marker-based motion capture, and it seems that our markerless motion capture system could be used especially as a skiing coaching tool. Cross-country skiing is developing all the time, with new modifications of subtechniques such as the “double-push” skating technique [33]. Race speeds have also increased, and this increases the importance of both skiing technique and skiing equipment [24]. Our markerless motion capture system presents interesting opportunities for cross-country skiing technique research and coaching. The markerless system combined with Coachtech system provides the results automatically [26], so changes in skiing technique could be viewed during training. There are several possibilities for using the markerless system from the coaching perspective. For example, the system provides possibilities for analyzing several parameters connected to technique in addition to joint angles, like body position and forward lean. Typically, the coach provides some instructions to the skier to change the skiing technique during training. With this markerless system, it can be determined whether the athlete has modified their skiing technique accordingly. In addition, it can be monitored how these technique changes influence, for example, the skiing economy and/or maximal skiing performance. In addition, this approach provides opportunities for capturing large amounts of skiing technique data. In the future, we could develop markerless models for other skating techniques (G4 and G5) and classic skiing.

Previous studies have provided information about propulsive forces during skiing [34,35]. In addition, the analysis of propulsive forces is important in the biomechanics of cross-country skiing, because it significantly affects the skiing speed. In the future, our markerless motion capture system could be used simultaneously with force sensors, for example, with the recently developed force measurement roller skis [36]. Thus, a more comprehensive analysis of the biomechanics of cross-country skiing could be obtained without additional equipment or markers.

In conclusion, we present and validate a markerless motion capture system that is low cost and easy to use for researchers and coaches. This markerless motion capture system could be used as a real-time coaching tool to monitor and improve skiing techniques.

## Figures and Tables

**Table 1:** Protocol of roller skiing trials, including the skiing technique, speed of treadmill, and angle of treadmill for each trial. Note that for G3, treadmill speeds differed for males (m) and females (f).

Trial	Skiing Technique	Speed of Treadmill (km/h)	Angle of Treadmill (deg)
1	G1	8	5
2	G1	6	8
3	G1	8	5
4	G1	6	8
5	G3	12 f/14 m	2
6	G3	18 f/20 m	2
7	G3	24 f/26 m	2

**Table 2:** Mean difference, standard deviation, and 95% limits of agreement for joint centers and toe placements.

Detected Point	Skiing Technique	Mean Difference (Bias) [mm]	±SD [mm]	95% LoA Lower/Upper
Wrist	G1	40.3	30.1	40.4/40.5
	G3	38.3	22.6	38.1/38.5
Elbow	G1	33.6	28.9	33.3/33.8
	G3	29.5	17.5	29.4/29.7
Shoulder	G1	26.6	19.7	26.4/26.8
	G3	24.0	14.1	23.9/24.1
Hip	G1	31.9	14.9	31.8/32.0
	G3	31.3	16.1	31.2/31.5
Knee	G1	30.5	16.6	30.4/30.7
	G3	30.2	20.2	30.0/30.4
Ankle	G1	31.5	17.0	31.4/31.6
	G3	32.4	22.9	32.2/32.6
Toe	G1	40.8	21.3	40.6/41.0
	G3	39.4	29.7	39.1/39.7

**Table 3:** Mean difference, standard deviation, 95% limits of agreement, RMSE, Pearson correlation, and intra-class correlation coefficient for joint vector angles.

Angle	Skiing Technique	Mean Difference (Bias) [deg]	±SD [deg]	95% LoA Lower/Upper	RMSE [deg]	r Pearson Correlation	*p*-Value	ICC [Mean (95% Confidence Interval)]
Elbow	G1	0.99	7.16	0.94/1.06	7.23	0.96	<0.001	0.96 (0.95/0.96)
	G3	0.60	5.69	0.55/0.66	5.72	0.97	<0.001	0.97 (0.96/0.97)
Shoulder	G1	3.69	3.84	3.66/3.72	5.33	0.94	<0.001	0.92 (0.87/0.97)
	G3	2.71	3.76	2.68/2.74	4.64	0.96	<0.001	0.97 (0.95/0.98)
Hip	G1	1.08	4.06	1.05/1.12	4.21	0.94	<0.001	0.94 (0.92/0.95)
	G3	0.74	3.48	0.72/0.78	3.56	0.97	<0.001	0.97 (0.96/0.97)
Knee	G1	1.15	3.06	1.13/1.18	3.27	0.96	<0.001	0.95 (0.93/0.97)
	G3	0.82	2.89	0.80/0.85	3.01	0.96	<0.001	0.96 (0.95/0.97)
Ankle	G1	−2.47	4.31	−2.51/−2.44	4.96	0.82	<0.001	0.77 (0.59/0.86)
	G3	−2.03	4.23	−2.07/−1.99	4.69	0.84	<0.001	0.80 (0.68/0.86)

## Data Availability

The data are not publicly available because ethical restrictions were placed upon this data. In the informed consent form that was approved by the Research Ethical Committee, we stated that all data are confidential and will not be provided to third parties. Since it may be possible to identify individuals even after the anonymization of these data, our data cannot be shared publicly. Possible requests for limited data should be sent either to petra.l.m.torvinen@jyu.fi, to vesa.linnamo@jyu.fi, or to the Secretary of the University of Jyvaskyla Ethical Committee at secretary-ethicomm@jyu.fi.

## Appendix A

The marker set that was used in the evaluation study is shown in Table A1.

**Table A1:** Names, definitions, and positions for markers.

Marker Name	Definition	Position on Participant/Skiing Equipment
OFFSET	Right back	Anywhere over the right scapula
LSHO	Left shoulder	On the acromioclavicular joint
LSHF	Left shoulder front	Humerus lesser tubercle
LSHB	Left shoulder back	Same position as LSHF on the posterior side
LUPA	Left upper arm	On the upper lateral 1/3 surface of the left arm (placed asymmetrically with RUPA)
LELB	Left elbow	On the lateral epicondyle
LELBM	Left elbow medial	On the medial epicondyle
LFRM	Left forearm	On the lower lateral 1/3 surface of the left forearm (placed asymmetrically with RFRM)
LWRA	Left wrist marker A	At the thumb side of a bar attached to a wristband on the posterior of the left wrist, as close to the wrist joint center as possible. Loose markers can be used, but for better tracking of the axial rotations, a bar is recommended.
LWRB	Left wrist marker B	At the little finger side of a bar attached to a wristband on the posterior of the left wrist, as close to the wrist joint center as possible. Loose markers can be used, but for better tracking of the axial rotations, a bar is recommended.
RSHO	Right shoulder	On the acromioclavicular joint
RSHF	Right shoulder front	Humerus lesser tubercle
RSHB	Right shoulder back	Same position as RSHF on the posterior side
RUPA	Right upper arm	On the lower lateral 1/3 surface of the right arm (placed asymmetrically with LUPA)
RELB	Right elbow	On the lateral epicondyle approximating the elbow joint axis
RELB	Right elbow	On the lateral epicondyle
RFRM	Right forearm	On the lower lateral 1/3 surface of the right forearm (placed asymmetrically with LFRM)
RWRA	Right wrist marker A	At the thumb side of a bar attached symmetrically with a wristband on the posterior of the right wrist, as close to the wrist joint center as possible
RWRB	Right wrist marker B	At the little finger side of a bar attached symmetrically with a wristband on the posterior of the right wrist, as close to the wrist joint center as possible
RPSI	Right PSIS	Right posterior superior iliac spine (immediately below the sacroiliac joints, at the point where the spine joins the pelvis)
RASI	Right ASIS	Right anterior superior iliac spine
LPSI	Left PSIS	Left posterior superior iliac spine (immediately below the sacroiliac joints, at the point where the spine joins the pelvis)
LASI	Left ASIS	Left anterior superior iliac spine
LTHI	Left thigh	Over the lower lateral 1/3 surface of the left thigh
LKNE	Left knee	On the flexion–extension axis of the left knee
LTIB	Left tibia	Over the lower 1/3 surface of the left shank
LANK	Left ankle	On the lateral malleolus along an imaginary line that passes through the transmalleolar axis
LHEE	Left heel	On the calcaneus at the same height above the plantar surface of the foot as the toe marker
LTOE	Left toe	Over the second metatarsal head, on the mid-foot side of the equinus break between forefoot and mid-foot
RTHI	Right thigh	Over the upper lateral 1/3 surface of the right thigh
RKNE	Right knee	On the flexion–extension axis of the right knee.
RTIB	Right tibia	Over the upper 1/3 surface of the right shank
RANK	Right ankle	On the lateral malleolus along an imaginary line that passes through the transmalleolar axis
RHEE	Right heel	On the calcaneus at the same height above the plantar surface of the foot as the toe marker
RTOE	Right toe	Over the second metatarsal head, on the mid-foot side of the equinus break between forefoot and mid-foot
LKNEM	Left knee medial	Left medial femur condyles
RKNEM	Right knee medial	Right medial femur condyles
LANKM	Left ankle medial	Left medial malleolus
RANKM	Right ankle medial	Right medial malleolus
LSKI1	Left Ski 1	On the front side of the binding of the left roller ski, on the left edge, on the front side of LSKI2
LSKI2	Left Ski 2	On the front side of the binding of the left roller ski, on the left edge, on the back side of LSKI1
LSKI3	Left Ski 3	On the front side of the binding of the left roller ski, on the right edge, placed asymmetrically with LSKI1
RSKI1	Right Ski 1	On the front side of the binding of the right roller ski, on the right edge, on the front side of RSKI2
RSKI2	Right Ski 2	On the front side of the binding of the right roller ski, on the right edge, on the back side of RSKI1
RSKI3	Right Ski 3	On the front side of the binding of the right roller ski, on the left edge, placed asymmetrically with RSKI1
LPOLE1	Left Pole 1	At the top of the left pole, below the handle of the pole
LPOLE2	Left Pole 2	In the middle of the left pole, below LPOLE1
LPOLE3	Left Pole 3	At the bottom of the left pole, below LPOLE2
RPOLE1	Right Pole 1	At the top of the right pole, below the handle of the pole
RPOLE2	Right Pole 2	In the middle of the right pole, below RPOLE1
RPOLE3	Right Pole 3	At the bottom of the left pole, below RPOLE2
